# The exosome collection

**DOI:** 10.1186/s11658-025-00719-5

**Published:** 2025-03-24

**Authors:** Steven R. Goodman

**Affiliations:** Department of Pediatrics, College of Medicine, University of Tennessee Health Science Center, Memphis, TN 38163 USA

*Cellular and Molecular Biology Letters* (CMBL) has assembled a Collection of its recently published primary research and review articles on the important topic of exosomes and their roles in disease etiology, diagnosis and monitoring of progression, therapeutics, and current and future applications in regenerative medicine. Extracellular vesicles are released from the surface of all cells and fall into two major categories that can be distinguished based on their mechanism of exit from the cell of origin; size distribution, and composition of constituents: ectosomes and exosomes. Ectosomes are formed by evagination of the plasma membrane resulting in blebbing and the release of vesicles ranging in size from 50 nm to 1 μm, including microvesicles, microparticles, and large vesicles. Exosomes are of endosomal origin and have a size range of 40 nm to 160 nm, with 100 nm being the average. The formation of exosomes occurs in multiple steps including invagination of the plasma membrane, formation of an early sorting endosome, maturation into a late-sorting endosome, invagination of the late sorting endosome to form multivesicular bodies (MVBs) containing intraluminal vesicles (ILVs). The multivesicular endosomes can fuse with lysosomes resulting in degradation, or with the plasma membrane where the ILVs are released as exosomes.

Exosomes, originally believed to function in excretion of intracellular and membrane unwanted functional molecules, are now known to undergo a complex cargo sorting process that leads to their specific final content. They can be isolated from all body fluids including blood plasma, urine, saliva, lymph, cerebrospinal fluid, semen, breast milk and bronchoalveolar lavage fluid (BALF); and their composition studied by multiomic stack approaches including proteomics, transcriptomics, microRNAomics, genomic analysis, metabolomics and lipidomics. Secreted exosomes contribute to both normal physiological homeostasis and pathological processes by entering the interstitial space and then the circulation and delivering specific cargo to target cells via local paracrine or distal systemic endocrine-like signaling. The exosome signaling molecules enclosed within the biologic membrane, include specific proteins, lipids, mRNAs, miRNAs (small noncoding RNAs with about 22 nucleotides involved in gene silencing by binding to mRNA and inhibiting translation or leading to mRNA degradation), long noncoding RNAs (lncRNA) (> 200 nucleotides lacking open reading frames and controlling gene expression at the transcriptional and post-transcriptional level), and circular RNA (circRNA) that bind to miRNA inhibiting its targeting of mRNA and sometimes directly interacting with specific proteins. The biologic effects demonstrated in several of the Collection articles are attributed to specific exosome cargo miRNAs. Exosomes also contain nuclear single and double stranded genomic DNA and mitochondrial DNA. Each individual exosome will have its own unique set of cargo molecules which are defined by the cell type of origin, protein cargo sorting by post translational modifications including ubiquitination/deubiquitination and binding to ESCRT complexes, miRNA sorting based on short sequence motifs and specific sorting proteins, and the metabolic influence of cell cycle events, cellular stress and inflammation due to specific disease status. Exosome targeting and uptake is based on the proteins and ligands on the surface of the exosome and its target cell membrane. Exosome uptake by endocytosis can be clathrin dependent or independent. Exosomes can also be internalized by the target cell via micropinocytosis, phagocytosis and fusion mechanisms. Due to their size and cell surface composition, exosomes can also cross the blood brain barrier making them useful in the diagnosis and treatment of neurodegenerative diseases.

Due to the noninvasive ease of obtaining exosomes from body fluids and the stability of its encapsulated cargo, they have proven valuable in preclinical studies for the identification of biomarkers for diagnosis, progression and therapeutic response for many diseases, including their use in liquid biopsies for cancer detection. The application of exosomes to produce anti-cancer vaccines also shows great promise. They are also being tested in early investigational and observational clinical trials for the usefulness of candidate biomarkers; as cell-free therapeutic agents; and as drug delivery agents. Clinical Trials.Gov indicates that there have been 444 investigational and observational clinical trial studies using exosomes of which I76 are currently looking for participants and 131 are actively recruiting. The advantages of MSC exosomes versus MSCs in clinical trials is that they are less likely to illicit an immune response in allogeneic trials, can more effectively reach their target because of their small size including crossing the blood brain barrier, and the lower likelihood of aggregating in the lungs upon IV administration. The primary disadvantage of MSC exosomes are the challenges of isolating pure and consistent lots in large enough quantities. Successful GMP production of exosomes for clinical trials requires large-scale production, uniformity and high quality, standard effective storage conditions that maintains the surface characteristics and cargo, enrichment of the therapeutic signaling molecules, and biodistribution testing and specificity of targeting.

Exosomes from mesenchymal stem cells or mesenchymal stromal cells (MSCs) are involved in tissue and organ rejuvenation and regeneration, as well as normal cellular physiology and homeostasis. But exosomes derived from infected, or disease associated cells tend to mediate pathogenesis. We find examples of both situations in this Exosome Collection of articles which the CMBL Editorial Board hopes you enjoy reading. I list several recent relevant comprehensive exosome reviews for interested readers [1–4].
